# Promoting equity in immunization coverage through supply chain design in Pakistan

**DOI:** 10.12688/gatesopenres.13121.1

**Published:** 2020-04-06

**Authors:** Mariam Zameer, Nora Phillips-White, Olamide Folorunso, Rachel Belt, Hamidreza Setayesh, Naeem Asghar, Arshad Chandio

**Affiliations:** 1 VillageReach, Seattle, WA, USA; 2 Supply Division, UNICEF, Copenhagen, Denmark; 3 Gavi, the Vaccine Alliance, Geneva, Switzerland; 4 Expanded Program for Immunization (EPI), Federal Ministry of Health, Islamabad, Pakistan

**Keywords:** equity, immunization, supply chain, cold chain

## Abstract

To improve equity in immunization coverage, potent immunization products must be available in the communities in which low coverage rates persist. Most supply side investments are focused on replacing or establishing new health facilities to improve access to immunization. However, supply chain design must be improved to ensure that potent vaccines are available at all facilities to promote immunization equity. We used the supply chain design process in Pakistan as an opportunity to conceptualize how supply chains could impact equity outcomes. This paper outlines our approach and key considerations for assessing supply chain design as a contributing factor in achieving equitable delivery of immunization services.

We conducted a supply chain analysis based on sub-national supply chain and immunization coverage at district level. Supply chain metrics included cold chain coverage and distances between vaccination sites and storage locations. Immunization coverage metrics included the third-dose diphtheria- tetanus-pertussis (DTP3) vaccination rate and the disparity in DTP3 coverage between urban and rural areas. All metrics were analyzed at the district level. Despite data limitations, triangulation across these metrics provided useful insights into the potential contributions of supply chain to equitable program performance at the district level within each province. Overall, our analysis identified supply chain gaps, highlighted supply chain contributions to program performance and informed future health system investments to prioritize children unreached by immunization services.

## Introduction

A strategic target of the Global Vaccine Action Plan is to achieve 90% national immunization coverage in all countries and 80% in each district by 2020 ^1^. Although global coverage has improved steadily since 1980, beginning in 2009 progress stagnated and it is unlikely these targets will be met by the end of 2020 ^2– 4^. Furthermore, inequities in immunization, or “avoidable differences in immunization coverage between population groups that arise because barriers to immunization among disadvantaged groups”, appear to be contributing to stagnation in coverage rates ^5^. Under-immunized children are more likely to be of lower socioeconomic status than fully immunized children and are geographically clustered in marginalized communities, such as urban poor, remote rural, mobile populations and communities affected by conflict, resulting in a higher burden of vaccine-preventable disease in these communities ^5– 14^. In order to continue making gains in equitable immunization coverage, interventions need to identify and specifically target these communities ^5, 6, 9, 13, 15, 16^.

As part of the effort to improve equity in immunization coverage, immunization products must be available and of sufficient potency in the communities in which low coverage rates persist ^4, 5^. To achieve this, immunization supply chains should be tailored to address health system barriers to equity in service delivery such as vaccine stockouts, inadequate storage capacity, non-functional cold chain equipment (CCE), as well as difficult terrain and road conditions ^4, 17– 22^. However, there is limited research and evidence on how the design of supply chains can be tailored to deliver potent vaccines to all children.

Supply chain designs traditionally focus on cost-effectiveness and efficiency by streamlining distribution and storage to improve performance ^23^. However, efficiency approaches could miss marginalized and hard-to-reach communities, and not address inequity. This paper describes the process by which equity was considered during an immunization supply chain (iSC) design analysis in Pakistan through a collaboration between the Pakistan Expanded Program on Immunization (EPI) ^24^, the United Nations Children’s Fund (UNICEF) ^25^, Gavi, the Vaccine Alliance ^26^, and VillageReach, an international non-profit that transforms health care delivery to reach everyone ^27^. The analysis identified disparities between districts by assessing supply chain, and immunization equity. This approach allowed stakeholders to consider placement of CCE, new warehouses or distribution network options to increase equity in immunization coverage.

## Methods

### Setting

Pakistan had an annual birth cohort of over 6.9 million children in 2017-18 with 6% of the world’s under-immunized children ^2, 28^. Currently, the Pakistan EPI is focusing on expanding routine immunization services, reaching every child with vaccines, and, as Pakistan’s Gross National Income per capita continues to grow, they will transition from Gavi financial support ^24, 29, 30^. As part of this strategy, which includes improving supply chain performance, an analysis was completed in 2018 to map the current supply chain structure and recommend changes to optimize Pakistan’s distribution network ^31^. We used LLamasoft’s Supply Chain Guru ^©^ modeling tool ^32^ to assess alternative supply chain designs defined by the Government of Pakistan, and supported by VillageReach, UNICEF, Gavi, and LLamasoft. Primary data such as vaccine demand, inventory policies, storage capacity, transport capacity, operating costs, and list of sites were collected from federal and provincial EPI. The modeling tool determined the optimal supply chain configuration for provinces to minimize costs while maintaining high levels of product availability. While the tool provides the most efficient solution for a supply chain system overall, it does not consider disparities and inequities within this system, such as between districts. Stakeholders in Pakistan, including the government, were highly interested in risk to vaccines and equity of service distribution, given such a large under-immunized population. From an immunization equity perspective, it is crucial to understand disparities in immunization coverage between and within districts in order to develop targeted strategies for under-immunized communities ^13^. Hence, VillageReach used Microsoft Excel to assess the data collected from primary and secondary sources to assess equity in the immunization supply chain.

### Assessing equity in Pakistan’s immunization supply chain

To provide further insights about the relationship between supply chain and equity in immunization coverage, we analyzed district-level metrics in 114 districts for 6,400 health facilities from the provinces of Balochistan, Khyber Pakhtunkhwa (KP), Punjab, and Sindh, and Islamabad that cover over 90% of Pakistan’s population. Supply chain metrics were aggregated from the collected data and modeling outputs, and we obtained district-level immunization coverage metrics from Pakistan Social and Living Standards Measurement survey (PSLM) ^33^. The PSLM was the only data source with immunization coverage rates at the district level that was available to us at the time of analysis. Although the PSLM has some methodological limitations, other data sources, including the Pakistan Demographic and Health Survey and the Multiple Indicator Cluster Surveys, were either not available for all provinces at the time of this analysis or were not representative at the district level ^34, 35^. We consulted with the Pakistan EPI and UNICEF, and determined PSLM was acceptable for the analysis.

### Supply chain metrics

**Figure 1.**
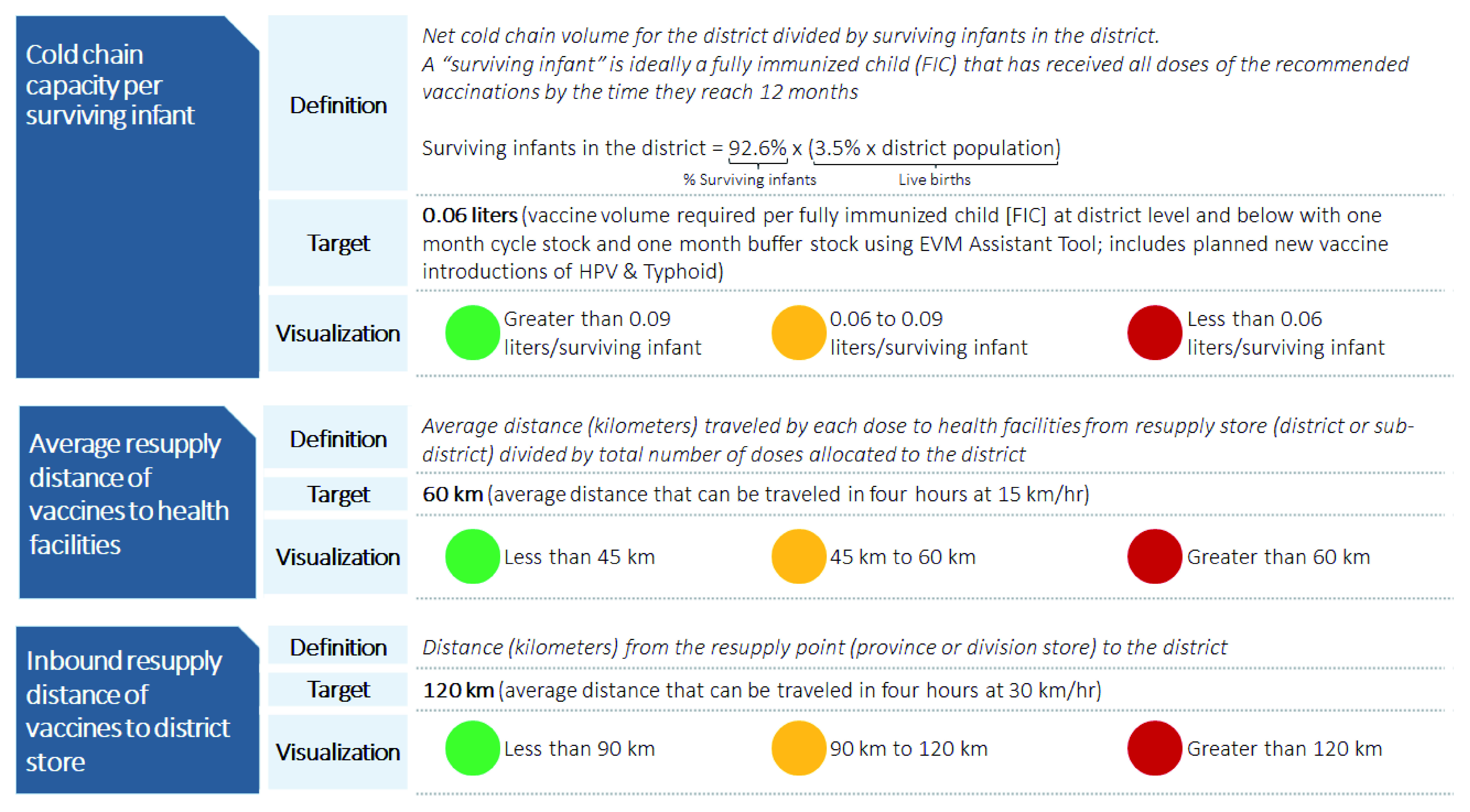
Three supply chain metrics to assess equity in supply chain design. Red - Indicator is outside range of the target; Yellow - Indicator is at the target range; Green - Indicator is well within the target.

We determined the appropriate supply chain metrics to include in our analysis by relying on data available to us and logical or evidence-based link to equity in immunization. Targets were set for each metric to contextualize districts’ supply chain performance, highlighting specific areas for decision-makers to consider during redesign. VillageReach, Gavi, and UNICEF consulted with the Pakistan EPI to develop appropriate targets for each indicator specific to Pakistan’s country context. The following sections describe each metric and our process to determine appropriate targets. Figure 1 summarizes the three supply chain metrics.

***Supply chain metric 1: Cold chain capacity per surviving infant at district and below.*** This metric assesses the cold storage capacity available to store vaccines at 2–8°C. Lack of functional cold chain constrains vaccine availability and can negatively impact vaccine potency, which limits immunization coverage rates ^12, 17, 36^. For our analysis, we defined cold chain capacity per surviving infant at the district level and below as the sum of the volume of cold chain storage available at the district stores, sub-district stores, and health facilities, divided by the projected number of infants in the district expected to survive to their first birthday in a one-year period, as shown in Figure 1. This metric included existing cold chain equipment at the time of the analysis plus equipment planned for deployment in 2018.

To understand whether cold chain coverage was sufficient, we used the World Health Organization (WHO) Effective Vaccine Management (EVM) tool to establish a target ^37^. This target represents the maximum volume needed to store all doses of the recommended vaccines required to fully immunize one child in Pakistan (assuming the child has survived to their first birthday and that products are resupplied once a month). Additionally, the target included Pakistan’s recommended “buffer stock” that should be available in case of spoilage, breakage, or unforeseen changes in demand. For Pakistan, this target was estimated at 0.06 liters of cold chain capacity per fully immunized child (FIC).

***Supply chain metric 2: Average resupply distance of vaccines to health facilities.*** This metric examines the distance that vaccines must travel to a health facility from the store resupplying it in the supply chain. Health facilities far from resupply locations may be at risk of low stock availability, and so low immunization coverage, because these distances may make the supply chain less responsive to outbreaks, emergency orders, or adverse weather. Finally, when vaccines must travel further to reach health facilities, they are more likely to be exposed to temperatures that put them at higher risk of spoilage and long distances can be a barrier for vaccinators for picking up supplies, which may lead to stockouts ^38^.

For each district, we calculated this metric by taking the weighted average of the distance from each health facility in the district to its resupply store, which was based on the estimated number of doses required for each health facility to serve its target population. We set a target of 60 kilometers as a feasible trip for a vaccinator to travel from a health facility to its resupplying store and return in one business day, based on the average travel speeds reported during primary data collection across all provinces. While this target does not account for variability in road conditions, it does serve as a point of comparison to identify districts where many facilities are far from resupply points.

***Supply chain metric 3: Inbound resupply distance of vaccines to district store.*** We developed this metric to understand supply chain configuration at the intermediate levels of the supply chain, upstream from the health facility. When stockouts occur at the district level, service delivery is interrupted in 96% of cases ^39^. Additionally, longer resupply distances to districts could result in similar risks to vaccine quality and availability mentioned above. To that end, the inbound resupply distance to the district store measured the distance that vaccines must travel to reach the district from either a province or division store, depending on the province in Pakistan. Based on data collected from provinces, we determined that 120 kilometers was a feasible trip from a district store to its resupply point and back in one business day, and set the target accordingly.

### Immunization coverage metrics

Low coverage rates result from several factors in the health and social systems including, but not limited to, supply chain challenges ^11, 22, 40^. To explore the relationship between immunization coverage and supply chain configuration, our analysis focused on two coverage indicators, both collected from the PSLM.

***Immunization coverage metric 1: DTP3 coverage.*** This metric measures the percentage of children vaccinated with the third dose of the diphtheria-tetanus-pertussis (DTP3) vaccine based on a survey of a sample of households in each district ^33^. DTP3 coverage is widely accepted as a proxy for full immunization coverage ^8^. To compare districts relative to each other, we organized districts by percentile; the 90 ^th^ percentile for this metric corresponds to a DTP3 rate above 91%, and 70 ^th^ percentile indicates a DTP3 coverage rate above 72%.

***Immunization coverage metric 2: Disparity in DTP3 coverage between urban and rural areas.*** Although limited data below the district level were available for this analysis, the PSLM also provided an estimate of the difference in DTP3 coverage between the urban and rural areas in each district ^33^. Districts with large urban-rural gaps in immunization coverage may face disparities in supply chain performance between urban and rural areas, such as long resupply distances to rural areas ^14^. Again, we organized districts by percentile to facilitate comparisons, with districts in the 90 ^th^ percentile, indicating an urban-rural gap of less than 7 percentage points, and the 70 ^th^ percentile indicating an urban-rural gap of less than 22 percentage points.

## Results

### Applying an equity lens for supply chain design decision-making

Stakeholders used the results from our analysis to decide which districts to prioritize for changes to the supply chain, comparing the metrics for the current supply chain to the proposed design. See *Extended data* for the immunization supply chain equity metrics ^47^. Although the analysis did not reveal an overarching trend or pattern between the chosen supply chain metrics and DTP3 coverage, the results highlighted districts with high under-immunized populations and with disparities in urban-rural coverage that should be prioritized for supply chain interventions. The lack of clear trends is expected because many variables affect immunization coverage rates, including but not limited to supply chain, which were beyond the scope of our analysis ^41^. Pakistan is one of the three countries where polio is endemic, and reported 135 wild polio cases in 2019 according to the Global Polio Eradication Initiative. The polio initiative is a key way for Pakistan to identify zero-dose children who have not received immunization and districts with the most under-served populations that need specific supply and demand interventions. To add further context, districts classified as high priority for polio eradication by the Pakistan’s National Emergency Action Plan for Polio Eradication are highlighted throughout the analysis ^42^.

### Comparing cold chain capacity and immunization coverage

**Figure 2.**
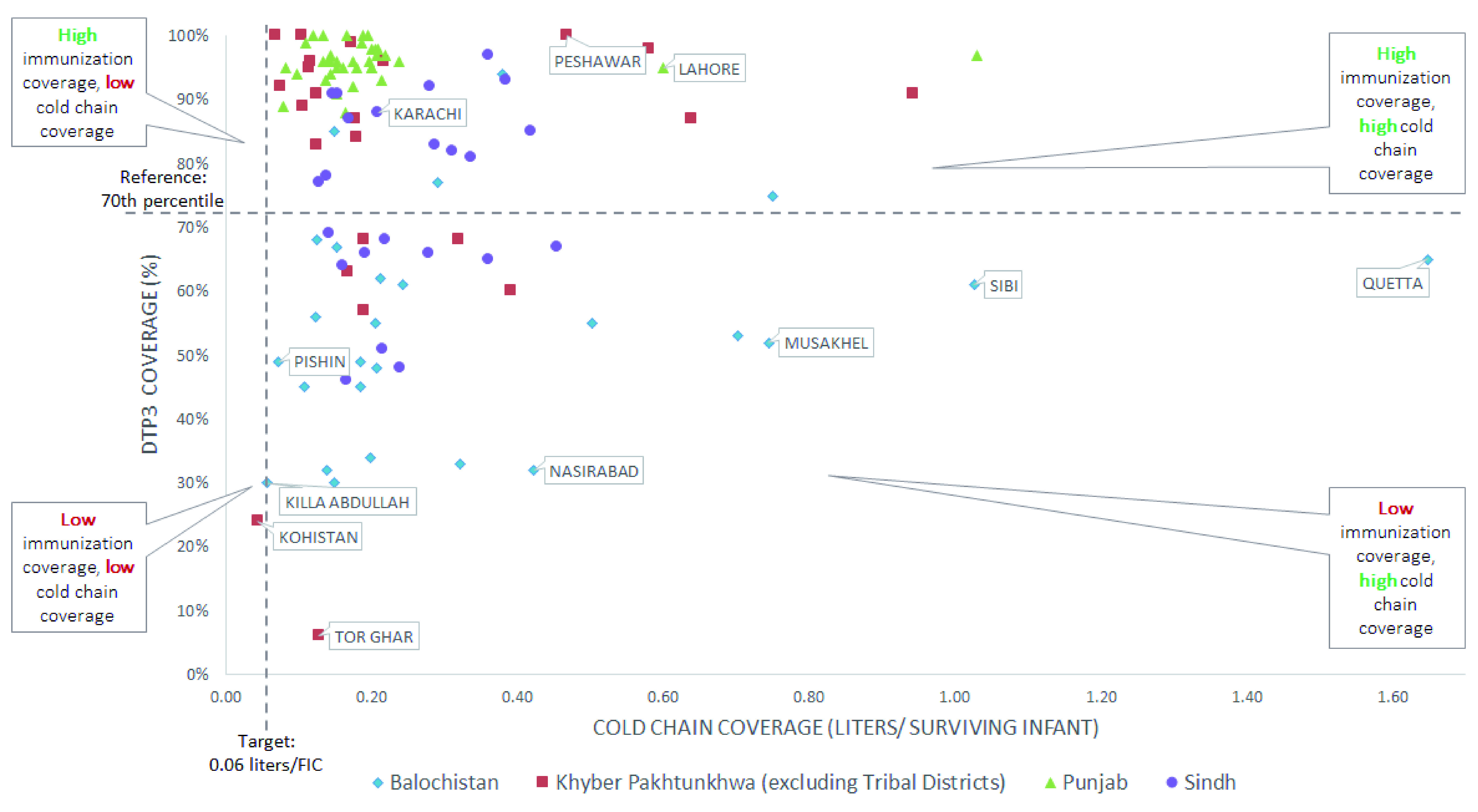
Cold chain capacity per surviving infant against immunization coverage for all provinces.

Our analysis indicated that the relationship between immunization coverage and cold chain capacity is ambiguous, as there are districts with cold chain capacity above the target but with low immunization coverage. Figure 2 shows several districts in Sindh, KP, and Balochistan, including Quetta, a high-priority polio district, have low immunization coverage rates, but cold chain capacity above the target. This indicates that factors aside from cold chain are likely leading to low coverage rates. We also highlighted districts with high disparities in urban-rural coverage rates, which might be a result of disparities in cold chain distribution within the district. A helpful next step would be to assess cold chain at each health facility, especially certain rural areas, to understand disparities within districts. Still, there are no districts with high immunization coverage and cold chain capacity below the target, reflecting that cold chain is a minimum condition needed to make potent vaccines available. We identified a group of districts with low immunization coverage and cold chain capacity near or below the target ( Figure 2), including Kohistan district in KP and two high-priority polio districts in Balochistan, Killa Abdullah and Pishin. Government stakeholders took note of these findings, and plan to prioritize these districts for receiving new CCE in an upcoming round of cold chain deployment through the Cold Chain Equipment Optimization Platform (CCEOP).

### Comparing weighted average resupply distance to health facilities and immunization coverage

**Figure 3.**
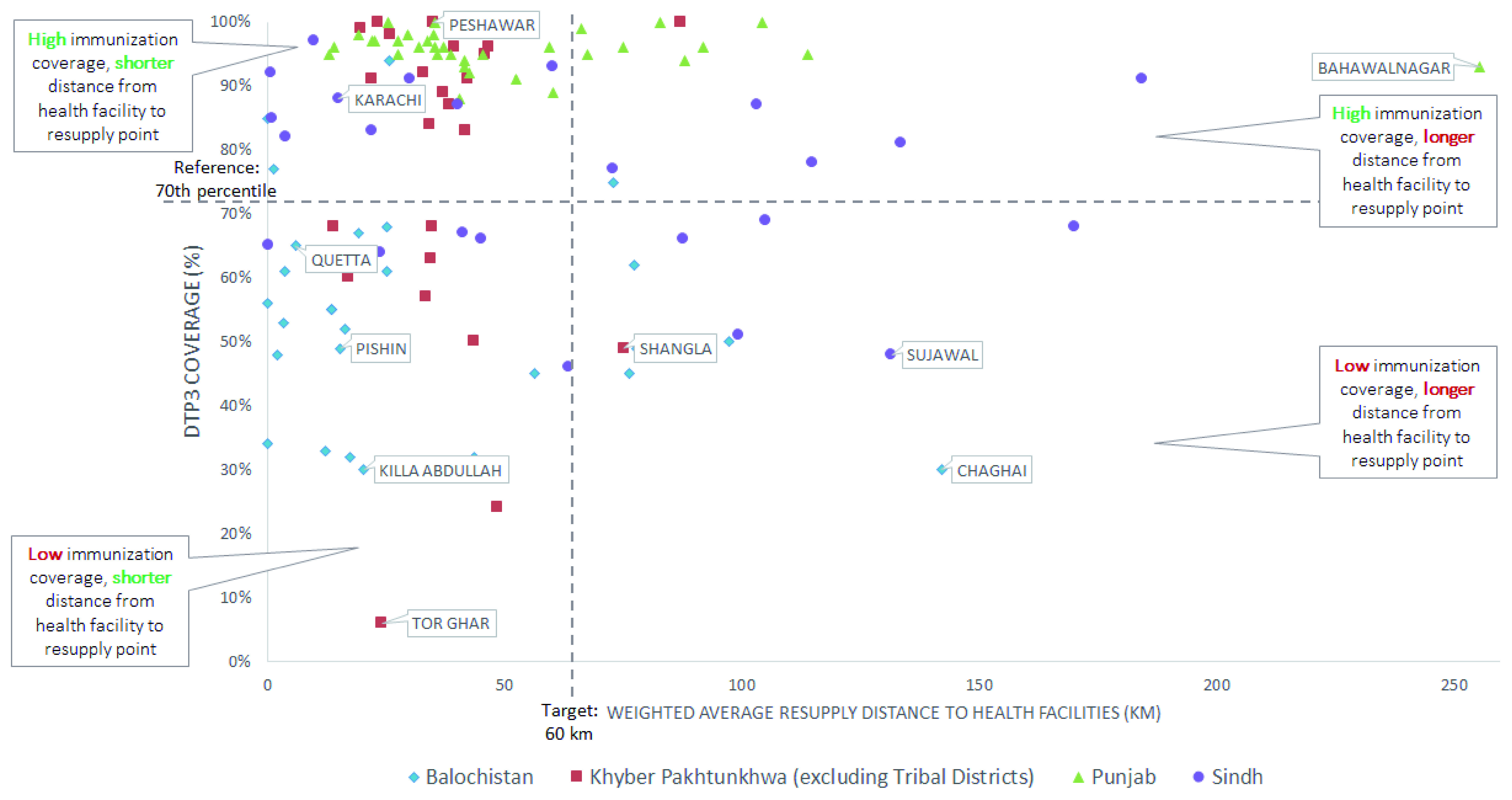
Weighted average resupply distance to health facilities against third-dose diphtheria-tetanus-pertussis (DTP3) for all provinces.

Examining the distance from health facilities to their resupply points, alongside immunization coverage, helped decision-makers identify districts where resupply distances should be reduced, as shown in Figure 3. Many districts in KP and almost all districts in Punjab had resupply distances for health facilities within the target and high coverage rates, illustrating shorter resupply distance may be a contributing factor to high coverage. On the other hand, in most districts in Balochistan resupply distances were also within the target; however, immunization coverage rates were low, indicating that other factors may underpin low coverage. The results for Sindh were varied, with no apparent trends. A helpful step would also be to assess individual resupply distances for health facilities in rural areas, especially where there is large disparity in urban-rural immunization coverage rates.

**Figure 4.**
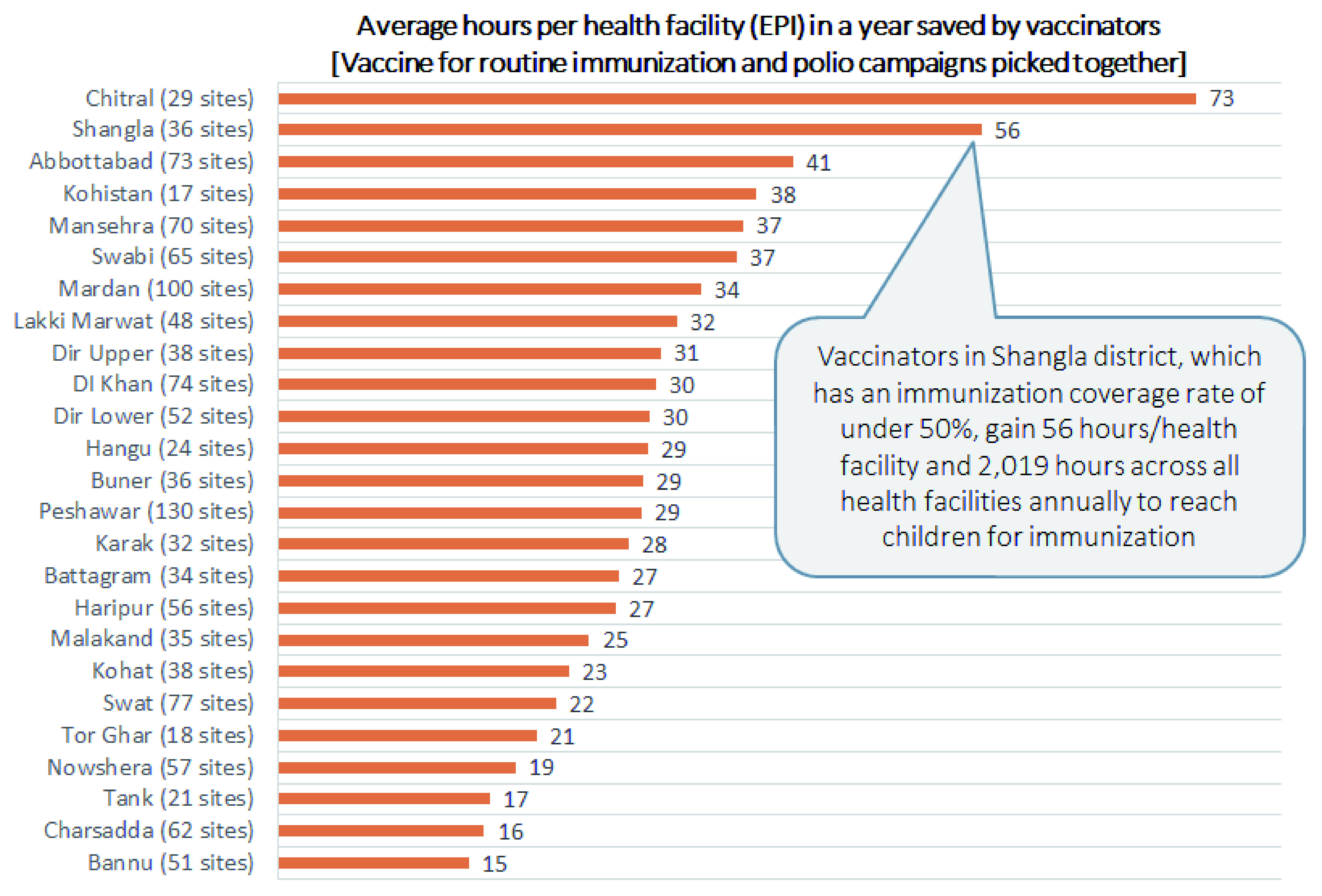
Average hours saved by each vaccinator in a year per health facility from direct delivery in Khyber Pakhtunkhwa province (excluding Tribal Districts).

To improve product availability in health facilities that are far from their resupply points, stakeholders in KP considered changing from the current distribution system in which vaccinators fetch products, to direct delivery of stock to health facilities from districts ^23^. This strategy consolidates responsibility for logistics at a higher level of the supply chain where a smaller group of workers can receive specialized training to avoid mishandling vaccines during transit. Furthermore, direct delivery allows vaccinators at facilities more time to provide immunization services ^43^. For example, Figure 4 shows vaccinators in Shangla district, in which less than half of children have received the DTP3 vaccine, could save 2,019 hours annually, which could be spent on providing immunization services. This is especially critical in areas with low rural coverage rates where the resupply distances may be very long.

### Comparing inbound resupply distance of vaccines to district stores and immunization coverage

**Figure 5.**
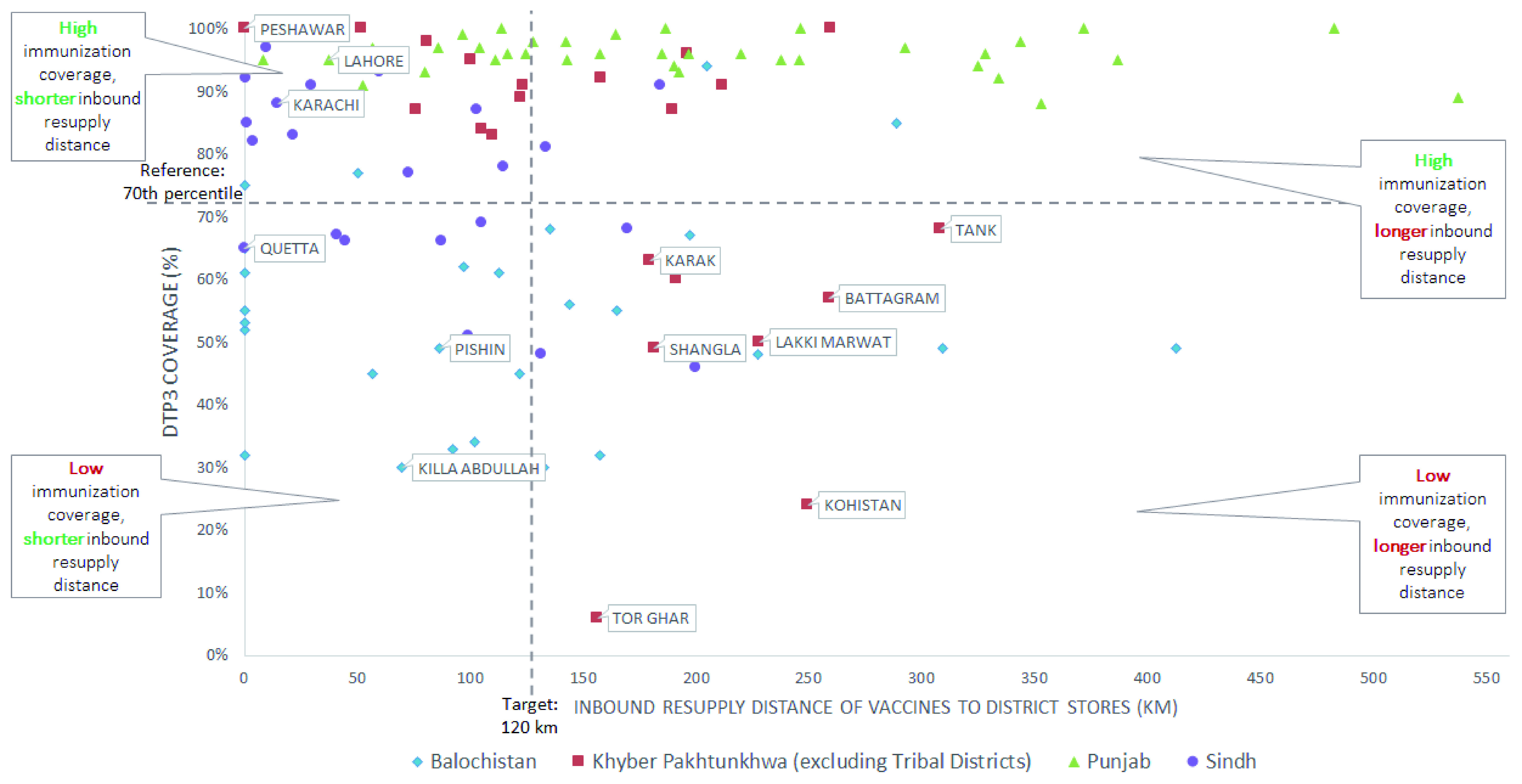
Inbound resupply distance of vaccines to district stores for all provinces against third-dose diphtheria-tetanus-pertussis (DTP3) for all provinces.

**Figure 6.**
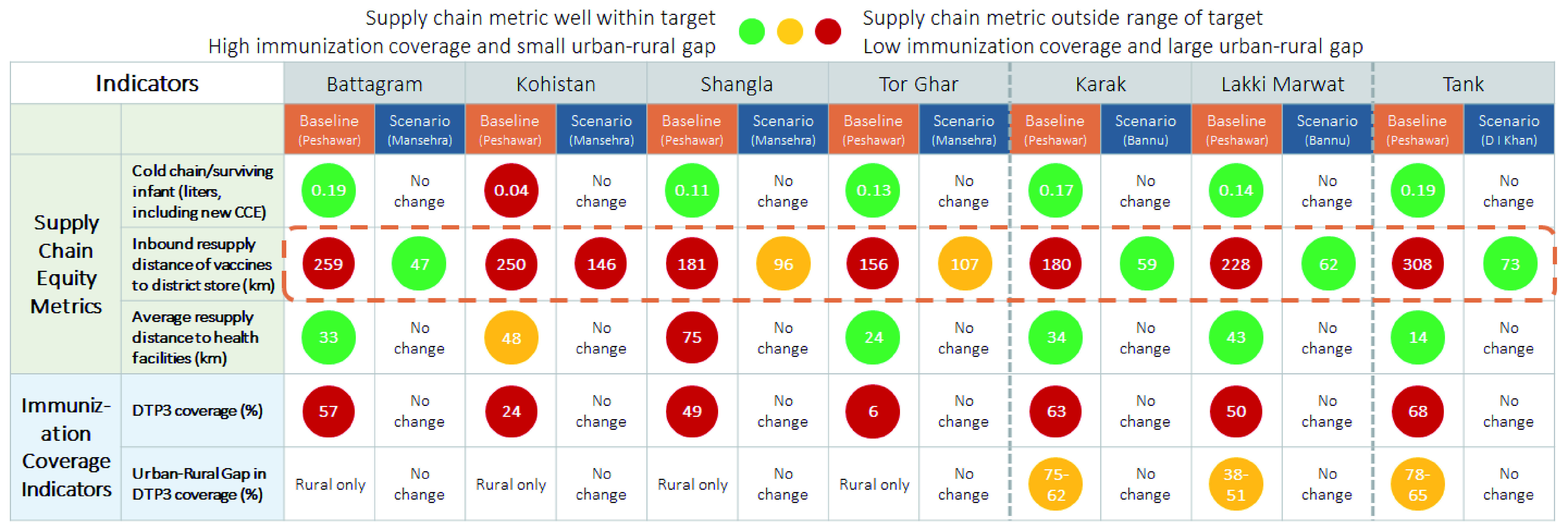
Changing sourcing would reduce resupply distance of district stores in KP (excluding Tribal Districts) improving reliability and responsiveness of supply chain, and contributing to improved immunization coverage.

The resupply distance for districts in the analysis ranged from 4 km to 538 km, as shown in Figure 5, but no notable relationships with immunization coverage are apparent. However, results were useful to stakeholders and provided further insights when considering supply chain design changes at the district level. For example, Figure 6 shows that changing a resupply store location in KP would reduce resupply distances to seven districts with very low rates of immunization coverage (Battagram, Kohistan, Shangla, Tor Ghar, Karak, Lakki Marwat, and Tank).

## Discussion

Our approach represents an important first step in using supply chain analysis to drive equitable design. Still, the analysis was constrained by several limitations that should be addressed in future iterations.

### Improving data availability to extend analysis to the community level

Due to constraints in the dataset, the district was the lowest level of analysis possible in Pakistan, yet inequities in immunization often exist between communities within districts ^13^. For example, we know that the distance that a person must travel to receive vaccinations is associated with coverage ^12, 13^, yet data was not available on the distances between health facilities and the communities they serve. In addition, due to security concerns, there is restriction on collecting geospatial information in Pakistan, which limited our ability to locate facilities and calculate such distances.

Lack of data at lower tiers of the health system is not unique to Pakistan; a recent assessment showed that 74 countries (38% of 194 countries worldwide) do not report sub-national immunization data ^2^, let alone at the district, health facility or community level. Similarly, high-quality supply chain data is not consistently available for lower levels of the health system ^44^. For more specific analysis to identify inequities, countries should assess cold chain capacity and resupply distance for each health facility in relation to immunization coverage. Further, supply chain metrics such as stock availability, on-time/in-full deliveries, and number of temperature excursions for CCE should be used for design. In order to improve equity in immunization, it is critical to have visibility into the supply chain challenges that must be overcome to ensure that potent vaccines are available in all communities.

### Developing metrics suited to urban areas

In urban areas, distances between points may be quite short but may take long to cover due to traffic, so resupply distance metrics from this analysis may not provide the full picture. However, even in densely populated urban areas, research shows that placement of facilities near low-income communities seems to be an important factor in urban areas to improving coverage. However, an alternative metric may be needed to show proximity of health facility to resupply to ensure availability of potent vaccines, such as time ^18, 19^. Additionally, lack of data on population in rapidly growing urban centers results in inadequate cold chain capacity to meet demand, leading to stockouts ^45^. As a result, urban-specific metrics should be developed and measured at the community-level to identify inequities within cities.

At the time of this analysis, detailed data on Pakistan’s urban poor was not available, although data from focus groups from a separate urban poor study by UNICEF highlighted lack of public health facilities in urban poor communities. However, these data did not identify names of nearest health facilities, so we could not generate specific recommendations to improve the supply chains. Similarly, the PSLM, which provided district-level immunization coverage data, aggregated the six districts in the mega-city of Karachi into a single sampling block, so we could not assess disparities in coverage rates in this major urban area. Recently Gavi and Pakistan EPI have been collecting data in Karachi to identify inequities in the distribution of cold chain and other components of the immunization program.

### Implementing master facility lists to assess equity across data sources

A master facility list is critical to match data across different databases, which are created and managed by various partners. When health facility names cannot be matched across different sources, the ability to use available data and to assess cold chain capacity at the health facility-level is limited. In this analysis, VillageReach was only able to match names of 25% of the 8,000 public health facilities in Pakistan between multiple databases. To facilitate data analysis at the facility-level and understand inequities between communities, countries should develop a master facility list with unique identifiers.

## Conclusion

Often, the design of supply chains is based on cost-efficiency analysis; however, our approach presented in this paper provides guidance on applying an equity lens when making changes to the supply chain design. The analysis enabled decision-makers in Pakistan to consider alternative supply chain configurations in light of potential improvements to equity and coverage. Further, while most previous analysis has focused on inequities between provinces ^35^ or focused on a small sample of districts ^46^, this analysis moves towards a full-country analysis of immunization equity at the district level, which is a critical step to identifying districts which need closer analysis. Previous studies often focus on either provincial analysis which hides disparities or on a subset of districts in a country which does not provide the full picture.

Considering equity in addition to other dimensions such as cost and efficiency yields important benefits for wider planning. Many supply-side investments focus on the replacement and improvement of existing facilities, which does not improve the status quo in underserved areas. While the analysis demonstrated that the relationship between supply chain inputs and program performance is not always clear, supply chain redesign could potentially reduce stockouts and vaccine expiry, inform cold chain deployment in low coverage areas, and ultimately contribute to equitable immunization.

In future applications, this analysis must be adapted to the context in which it is being used by selecting appropriate and salient metrics. Many Gavi-eligible countries receive CCE through CCEOP, so metrics such as cold chain capacity per surviving infant can be used to systematically include equity in deployment. Country-specific targets must be calculated for each country. Furthermore, inequities in immunization coverage in urban areas are a priority in many countries, so urban-specific metrics need to be developed. Finally, product availability and potency is only one aspect of the service delivery, and supply chain design should seek appropriate linkages with other supply dimensions (e.g. financing, service delivery, etc.) and demand multi-dimensional interventions to impact program performance. Integrating equity considerations into the supply chain design process can encourage stakeholders to identify areas for holistic supply chain improvements that prioritize underserved and under-immunized populations.

## Data availability

### Underlying data

All data underlying the results are available as part of the article and no additional source data are required.

### Extended data

Figshare: Data_Pakistan_GatesOpenResearch. https://doi.org/10.6084/m9.figshare.12032106.v1 ^47^.

This file contains the immunization supply chain equity metrics for Pakistan produced in this study.

Extended data are available under the terms of the Creative Commons Attribution 4.0 International license (CC-BY 4.0).

## Disclaimer

The findings and conclusions contained in this report are those of the authors and do not necessarily reflect Gavi and UNICEF policies and positions.
