# Supplementary material for: Promoting equity in immunization coverage through supply chain design in Pakistan
Source: Gates Open Res. 2020 Apr 6;4:31. [Version 1] doi: 10.12688/gatesopenres.13121.1 (PMC7926263; doi:10.12688/gatesopenres.13121.1)
Supplement: Peer review history

# Peer review: Reviewer response for version 1

**DOI:** 10.21956/gatesopenres.14300.r30187

**Published:** 2021-03-08

**Authors:** Shawn T. Brown

**Affiliations:** 1 Pittsburgh Supercomputing Center, Carnegie Mellon University and the University of Pittsburgh, Pittsburgh, PA, USA; 2 Mellon College of Science, Carnegie Mellon University, Pittsburgh, PA, USA

The manuscript describes an effort in Pakistan amongst international partners to attempt improve equity in vaccine coverage through supply chain design. The authors have utilized data from the Pakistan supply chain and worked closely with in-country decision-makers to analyze data in an effort to find metrics that are correlated to identify items in the current design that could be manipulated to improve the cost of the supply chain and improve equitable coverage in the country. While I am excited about the work and think that many of the points in the paper are interesting, I think overall the manuscript has several flaws that would need to be addressed significantly before I believe it could be considered a complete contribution to the topic.

 Broadly, I highlight these below:

1. The authors present three metrics where they look for some correlation with DTP coverage. It is arguable that in all three cases, the metrics did not correlate very well coverage, which I would not expect them to in the first place. All three are entirely static metrics and do not holistically speak to the overall challenges that are associated with coverage (or even vaccine availability) at a location. The first metric, the amount of cold capacity per surviving infant at district and below to some extent will indicate that there is enough capacity for storing the vaccines, but just having functional storage capacity is only one piece of the puzzle. How stable is the power supply at the location? How often is the refresh rate of the vaccine delivery? What are the expected wastage rates at these facilities based on vaccine demand? The are just a few questions that arise from storage needs that this metric does not capture. If I am reading correctly, and the point is a bit confusing, the metric is defined as surviving infants per one year period. Please clarify this. Again, is this based on functioning cold chain equipment? 8C or freezer? And wastage will present a huge factor here, especially in rural areas where the number of doses administered may be 1 or two a day, which means that cold chain per surviving infant would have to be higher than in an area where there are multiple patients per day.
2. Both distance metrics, again, are very static measures and I am not surprised that they do not correlate to coverage. Again, distance is not necessarily a solid indicator of the quality of a supply route. The quality can vary quite a lot given urban vs. rural, traffic patterns, shipping vehicle, and frequency. This metric also does not speak at all to the cold chain capacity given on the vehicles that are transporting. So again, while I can see the metrics value, the overall design of the supply chain is not solely or significantly dependent on the distance.
3. There is software being used in the study, Llamasoft's Supply Chain Guru, of which I am familiar. It is a very nice package. Other than it being mentioned in the methods, I am not sure at all how the software was utilized in the study. The results presented seem to be from the spreadsheet that is provided as a supplement on FigShare (which I think is great that the authors provided and shared). If there were results or methodologies that this software was used for (which is not open source, and therefore the reviewer would need to purchase to obtain), they should be described.
4. There are no costs in this analysis. This should be straight forward given that the authors know the route distances, and if they know the route vehicles, it would add tremendously to the analysis. Again, just because a route is longer, doesn't mean it is less cost effective, as it may be a long route, with a vehicle that carries a lot of vaccine. I don't see how using the metric of just "shortening" trips immediately leads to an improved vaccine supply chain.
5. I am not sure at all how this study addresses equity. I found the one paragraph that speaks to this basically just says "Look at our data, these are places with low immunization coverage." Equity in immunization coverage goes beyond "this is an area of low immunization coverage", and has fundamental components of policy not covered in this analysis.
6. This reviewer finds the conclusions of the study follow from the analysis, in that they claim that one needs more data to gain a better picture of the supply chain. I agree, although while the lack of solid results in the analysis indicate there is a need for more data, this is not a result that I would say warrants publication.
7. As this is an open research submission, I would be remiss to talk of availability of data and software. I applaud the authors providing the spreadsheet that was used to create their scatter plots, indeed a lot of the synthesized data is in there. There is much of the data that went is mentioned in the study that is not available openly. I am aware that there are always sensitivities with country data, so I will leave it to the journal to decide as to the necessity of providing it.

Overall, while I am sure that the team that authored this paper is doing great and relevant work, I think the work here reads more as a high-level brief than an actual academic, methods paper. And I think that supply chain design is important, but the static metrics devised here, the lack of economic analysis, and results that merely show that the metrics are not adequate to find correlation to the actual issues of coverage make this manuscript as submitted not very rigorous or useful. It is a good start as a preliminary analysis, but further work should be done to actually provide the government with a real ability to explore and change the system to promote vaccine coverage and equity.

Is the rationale for developing the new method (or application) clearly explained?

Partly

Is the description of the method technically sound?

Partly

Are the conclusions about the method and its performance adequately supported by the findings presented in the article?

Partly

If any results are presented, are all the source data underlying the results available to ensure full reproducibility?

No

Are sufficient details provided to allow replication of the method development and its use by others?

No

Reviewer Expertise:

Vaccine supply chain modeling and redesign.

I confirm that I have read this submission and believe that I have an appropriate level of expertise to state that I do not consider it to be of an acceptable scientific standard, for reasons outlined above.

# Peer review: Reviewer response for version 1

**DOI:** 10.21956/gatesopenres.14300.r30188

**Published:** 2021-03-02

**Authors:** Kath O'Reilly

**Affiliations:** 1 Centre for Mathematical Modelling of Infectious Diseases, Department of Infectious Disease Epidemiology, Faculty of Epidemiology and Population Health, London School of Hygiene and Tropical Medicine, London, UK

It's nice to see something in cold chain logistics as this is an important and sometimes overlooked area. I would regard this analysis as a pilot study, as there wasn't really much that was convincing about the study. Consequently, I think the discussion should be more focused around what the researchers learnt and how researchers can move forward to make progress in this area. Data on vaccine stockouts and some sort of engagement with district level health practitioners would go a long way.

 Abstract:

- “potent immunization products” - replace with vaccines. PIP is a bit of a confusing term, and immunisation coverage is usually just the proportion of target product reached rather than a measure of whether they have an immunological response. Unless there’s a reason why ‘immunisation products’ would be anything other than vaccines, but I really cannot think of one, especially in the current setting. I don’t really understand why ‘potent vaccines’ is used as a term either - why not just vaccines? Your focus is delivery of vaccines, not whether they remain potent - e.g. There is nothing in the paper about cold chain.

Methods:

- This paper is described as a “method article” - what’s new here?
- Only looked at DTP3 coverage. To an extent this is okay, but if there is a specific DTP stockout this may bias the findings. It would be preferable to explore multiple antigens (eg. MCV).
- As well as cold chain ‘capacity’ can you provide details of how this was measured? It a physical thing, so was it measured by a team or was a survey sent out? This isn’t clear to me. Capacity can sometimes be as simple as a cold storage box, but sometimes far bigger. Did you also have information on the stock available in each health facility?
- For the health facility, what data are you using, e.g. Data provide by the MoH? How do you ascertain it’s functionality? For example is it open 7 days a week or something else? I’m being naive here because in some settings health centres don’t always precisely exist despite being there ‘on paper’. I’d also like clarification on the number of health centres per district and how the weighted average was calculated.
- Will you provide this valuable information on health centres for other researchers?
- Have you considered including health centres per 100,000 population or some similar measure - does this explain any of the differences in coverage?
- What is a district store and how is it different to a resupply point?

Results:

- Figure 2: It’s clear that the minimal target has been met in a majority of districts, and isn’t a ‘causal’ factor for poor coverage. What I find puzzling is why some districts have capacity that is orders of magnitude higher? I think you should provide reasons for this - I presume you have an idea?
- I don’t understand what ‘direct delivery’ means, please explain in more detail. If the resupply point is cut out, who and how is it picked from the higher point? It feels like Fig 4 is an over-simplification of a complex process - will there not be unintended consequences?
- You haven’t convinced me that supply chain is in any way (that you’ve measured) related to DPT coverage. Your caption for Fig 6 suggests that improving these would improve DPT coverage and I don’t think you can make that statement.

Discussion:

- I’m not convinced that having sub-district data would improve the analysis. I’m also confused how you can calculate distance from resupply to health facility but not health facility to population. Worldpop provides excellent data on population location and surely you can include in analysis? I think it would be better to write that exploring distance of populations to health facility would be an important next step.
- I agree with your statement about needing to know about stock availability, for me this is a critically missing piece of information in this analysis. I think you should be more explicit about what (important) factors were not included in this analysis, and what steps could be made to include them in the future.

Is the rationale for developing the new method (or application) clearly explained?

Partly

Is the description of the method technically sound?

Partly

Are the conclusions about the method and its performance adequately supported by the findings presented in the article?

Partly

If any results are presented, are all the source data underlying the results available to ensure full reproducibility?

Partly

Are sufficient details provided to allow replication of the method development and its use by others?

No

Reviewer Expertise:

Quantitative epidemiology, public health in LMICs, immunisation coverage

I confirm that I have read this submission and believe that I have an appropriate level of expertise to confirm that it is of an acceptable scientific standard, however I have significant reservations, as outlined above.

# Peer review: Reviewer response for version 1

**DOI:** 10.21956/gatesopenres.14300.r28754

**Published:** 2020-05-06

**Authors:** Michelle Seidel

**Affiliations:** 1 UNICEF, Copenhagen, Denmark

**1)** **Is the rationale for developing the new method (or application) clearly explained?**

- The Equity Reference Group views equity from 4 lenses gender, conflict, urban poor and remote rural settings. It is not clear how the methodology assessed these 4 settings other than subdividing between rural and urban sites.

**2) Is the description of the method technically sound?**

- Slide - The supply chain metrics do not provide the assumptions for the parameters or the targets. The average resupply distance will negatively affect remote rural populations.
- The paper assumes that vaccine availability and cold chain will lead to immunization. It does not address the demand side factors or other supply side factors such as the availability of human resources, transport availability, etc.
- SC Metric 1: The analysis at district level for cold chain capacity is not useful seeing that vaccination services are delivered at the health facility level. It is not clear whether the analysis disaggregates the data at this level.
- SC Metric 2: Does not consider Cold chain equipment and uses averages.
- SC Metric 3: Does not control for variances in stock policies.

**3)** **Are sufficient details provided to allow replication of the method development and its use by others? **

- Some of the assumptions and targets for the calculations are missing.

**4) If any results are presented, are all the source data underlying the results available to ensure full reproducibility?**

- It is not clear how the metrics were derived and how some of the results were calculated.

 

- Considering equity certainly brings a new dimension to supply chain design. Although I would be cautious to deem the exercise in Pakistan as supply chain design but rather iSC optimization as it did not consider the entire health supply chain, which would further equity goals under UHC.
- The deductions drawn from the analysis and the lack of data at service delivery level leads to inconclusive results. The use of averages, particularly for distances is problematic when analyzing equity and I would suggest a categorization of distances rather.
- The analysis of equity in conflict zones is another opportunity this paper could have addressed in Pakistan.
- With some revisions to the paper, I would be happy to recommend it for indexing.

Is the rationale for developing the new method (or application) clearly explained?

Partly

Is the description of the method technically sound?

Partly

Are the conclusions about the method and its performance adequately supported by the findings presented in the article?

Partly

If any results are presented, are all the source data underlying the results available to ensure full reproducibility?

Partly

Are sufficient details provided to allow replication of the method development and its use by others?

Partly

Reviewer Expertise:

Immunization supply chain

I confirm that I have read this submission and believe that I have an appropriate level of expertise to confirm that it is of an acceptable scientific standard, however I have significant reservations, as outlined above.

# Response

**Published:** 2020-05-28

**Authors:** Mariam Zameer

**1) Is the rationale for developing the new method (or application) clearly explained?**

- The Equity Reference Group views equity from 4 lenses gender, conflict, urban poor and remote rural settings. It is not clear how the methodology assessed these 4 settings other than subdividing between rural and urban sites.
  - *Thank you for highlighting this. We agree that equity can be looked at from many different perspectives. Urban and rural are geographic dimensions that we focused on, while gender and conflict are socio-economic dimensions. Many people living in urban poor areas can be internally displaced as part of conflict settings.*

 

*While socioeconomic and gender barriers are useful equity dimensions, these data points were not consistently available as disaggregated data from the district, sub-district and health facility level, as acknowledged in the paper. The suggestion is well-taken, and we acknowledge the different dimensions on equity in the introduction paragraph 1: “Under-immunized children are more likely to be of lower socioeconomic status than fully immunized children and are geographically clustered in marginalized communities, such as urban poor, remote rural, mobile populations and communities affected by conflict, resulting in a higher burden of vaccine-preventable disease in these communities”. The disaggregated from the census survey as well as urban slum mapping was not available at the time of the analysis and concluded after the system design analysis; these would provide useful data points for further research and analysis that were not covered under the current scope.*

**2) Is the description of the method technically sound?**

- Slide - The supply chain metrics do not provide the assumptions for the parameters or the targets. The average resupply distance will negatively affect remote rural populations.
  - *In the Discussion section, we acknowledge that average resupply distance is a more appropriate metric for remote rural areas. One of our recommendations moving forward is the need to develop specific metrics for urban areas or other equity lens. The assumptions for the targets are outlined in Figure 1 and section on “Supply Chain Metrics”, e.g. for Metric 2, we state that “we set a target of 60 kilometers as a feasible trip for a vaccinator to travel from a health facility to its resupplying store and return in one business day, based on the average travel speeds reported during primary data collection across all provinces.”*

 

*In the Discussion, we also acknowledged that averaging resupply distances across districts could potentially mask the longer distances by inclusion of a number of relatively shorter distances. However, this potentially doesn’t drastically affect one population than the other as the metric was applied to all Provinces/Areas. With further data made available, Provinces could be further divided into not only urban/rural (based on distance) but also time and road accessibility to health facilities, which will accommodate for additional peculiarities of remote rural/urban populations. This is recommended as a next step for further analysis.*

 

- The paper assumes that vaccine availability and cold chain will lead to immunization. It does not address the demand side factors or other supply side factors such as the availability of human resources, transport availability, etc.
  - *Agree with the reviewer that we do not look at the other supply or demand side factors, and this is outlined in the introduction and framing paragraph 2: “As part of the effort to improve equity in immunization coverage, immunization products must be available and of sufficient potency in the communities in which low coverage rates persist. To achieve this, immunization supply chains should be tailored to address health system barriers to equity in service delivery such as vaccine stockouts, inadequate storage capacity, non-functional cold chain equipment (CCE), as well as difficult terrain and road conditions. However, there is limited research and evidence on how the design of supply chains can be tailored to deliver potent vaccines to all children.”*

*  *


*This analysis has focused on supply chain design’s contributions to programmatic outcomes. One limitation is that the factors considered in this analysis are only few of the supply and demand factors that contribute to programme performance. Other factors such as HR availability and competence, data availability and use, health facilities opening times, awareness creation etc. have an impact on performance.’*

 

- SC Metric 1: The analysis at district level for cold chain capacity is not useful seeing that vaccination services are delivered at the health facility level. It is not clear whether the analysis disaggregates the data at this level.
  - *We acknowledge this as a limitation of the study due to lack of data that was available at the health facility level, and lack of integrated data systems making it impossible to match health facility data across different databases. This is highlighted in the Discussion section. The authors believe that this is not peculiar to Pakistan and that systematic sub-national supply chain and programmatic performance capture and reliability of such data is challenging in similar countries. This study is however an important step in moving the supply chain contribution to programme outcomes discussion forward. Our recommendation is that this analysis should be extended to the health facility and community level where data is available, as outlined in the Discussion section. For Pakistan, as a first step, we think it is still valuable to identify districts and then be able to deep-dive for the specific districts.*
- SC Metric 2: Does not consider Cold chain equipment and uses averages.
  - *Request to the reviewer to clarify, as this metric is related to resupply distance and not cold chain storage. No average was used.*
- SC Metric 3: Does not control for variances in stock policies.
  - *In Pakistan, there were no variances in stock policies for districts to the best of our knowledge.*

**3) Are sufficient details provided to allow replication of the method development and its use by others?**

- Some of the assumptions and targets for the calculations are missing.
  - *Please let us know the assumptions and targets that are missing, so we add them appropriately. The targets for all metrics and calculations are presented in the Supply Chain Metrics section, and in Figure 1.*

**4) If any results are presented, are all the source data underlying the results available to ensure full reproducibility?**

- It is not clear how the metrics were derived and how some of the results were calculated.
  - *Request to reviewer to provide more details, so we are able to add more specific details. The Supply Chain Metrics section outlines how each of these metrics was derived and validated and presented in Figure 1. The data for each of the metrics is also available with the paper.*

 

- Considering equity certainly brings a new dimension to supply chain design. Although I would be cautious to deem the exercise in Pakistan as supply chain design but rather iSC optimization as it did not consider the entire health supply chain, which would further equity goals under UHC.
  - *The approach to supply chain design is based on the direction from the government, in this case the Government of Pakistan. We assessed and recommended changes to supply chain design based on direction from the federal and provincial government. Network optimization for supply chains usually uses specific modeling tools, which were also used in Pakistan. However, the authors went further than looking at the modeling results to look at comprehensive supply chain design.*

*  *


*The specific distinction between SC design and iSC optimization is misapplied here. The analysis presented here considers the system’s design and applied an equity lens using three metrics. From experience in multiple countries, System Design analysis could be a country-wide/entire supply chain or a specific scope (e.g. last mile, outreaches, cold chain utilization etc.).*

 

- The deductions drawn from the analysis and the lack of data at service delivery level leads to inconclusive results. The use of averages, particularly for distances is problematic when analyzing equity and I would suggest a categorization of distances rather.
  - *Refer to above for clarification of the use of averages. Averages was used as a form of categorizing findings. We see this work as a Methods paper, to further the conversation on equity in this area. As this methodology is repeated in other countries and refined, we would anticipate seeing trends that are helpful.*
- The analysis of equity in conflict zones is another opportunity this paper could have addressed in Pakistan.
  - *This was not an ask by the government of Pakistan, and the data on this is not available.*

# Response

**Published:** 2020-09-15

**Authors:** Mariam Zameer

***Thank you to the reviewer for the detailed comments. We respond to each comment below to clarify information and limitations that were included and acknowledged in the paper. We request clarifications for the changes and revisions that are requested to recommend the paper for indexing. We hope that our response would alleviate the reviewer's concerns and fully approve the paper, so it is available for other countries to use and to improve our methodology.***

**1) Is the rationale for developing the new method (or application) clearly explained?**

- The Equity Reference Group views equity from 4 lenses gender, conflict, urban poor and remote rural settings. It is not clear how the methodology assessed these 4 settings other than subdividing between rural and urban sites.
  - *Response: Thank you for highlighting this. We agree that equity can be looked at from many different perspectives. Urban and rural are geographic dimensions that we focused on, while gender and conflict are socio-economic dimensions. Many people living in urban poor areas can be internally displaced as part of conflict settings.* *While socioeconomic and gender barriers are useful equity dimensions, these data points were not consistently available as disaggregated data from the district, sub-district and health facility level, as acknowledged in the paper. The suggestion is well-taken, and we acknowledge the different dimensions on equity in the introduction paragraph 1: “Under-immunized children are more likely to be of lower socioeconomic status than fully immunized children and are geographically clustered in marginalized communities, such as urban poor, remote rural, mobile populations and communities affected by conflict, resulting in a higher burden of vaccine-preventable disease in these communities”. The disaggregated from the census survey as well as urban slum mapping was not available at the time of the analysis and concluded after the system design analysis; these would provide useful data points for further research and analysis that were not covered under the current scope.*

**2) Is the description of the method technically sound?**

- Slide - The supply chain metrics do not provide the assumptions for the parameters or the targets. The average resupply distance will negatively affect remote rural populations.
  - *Response: In the Discussion section, we acknowledge that average resupply distance is a more appropriate metric for remote rural areas. One of our recommendations moving forward is the need to develop specific metrics for urban areas or other equity lens. The assumptions for the targets are outlined in Figure 1 and section on “Supply Chain Metrics”, e.g. for Metric 2, we state that “we set a target of 60 kilometers as a feasible trip for a vaccinator to travel from a health facility to its resupplying store and return in one business day, based on the average travel speeds reported during primary data collection across all provinces.”* *In the Discussion, we also acknowledged that averaging resupply distances across districts could potentially mask the longer distances by inclusion of a number of relatively shorter distances. However, this potentially doesn’t drastically affect one population than the other as the metric was applied to all Provinces/Areas. With further data made available, Provinces could be further divided into not only urban/rural (based on distance) but also time and road accessibility to health facilities, which will accommodate for additional peculiarities of remote rural/urban populations. This is recommended as a next step for further analysis.*
- The paper assumes that vaccine availability and cold chain will lead to immunization. It does not address the demand side factors or other supply side factors such as the availability of human resources, transport availability, etc.
  - *Response: Agree with the reviewer that we do not look at the other supply or demand side factors, and this is outlined in the introduction and framing paragraph 2: “As part of the effort to improve equity in immunization coverage, immunization products must be available and of sufficient potency in the communities in which low coverage rates persist. To achieve this, immunization supply chains should be tailored to address health system barriers to equity in service delivery such as vaccine stockouts, inadequate storage capacity, non-functional cold chain equipment (CCE), as well as difficult terrain and road conditions. However, there is limited research and evidence on how the design of supply chains can be tailored to deliver potent vaccines to all children.”* *This analysis has focused on supply chain design’s contributions to programmatic outcomes. One limitation is that the factors considered in this analysis are only few of the supply and demand factors that contribute to programme performance. Other factors such as HR availability and competence, data availability and use, health facilities opening times, awareness creation etc. have an impact on performance.’*

- SC Metric 1: The analysis at district level for cold chain capacity is not useful seeing that vaccination services are delivered at the health facility level. It is not clear whether the analysis disaggregates the data at this level.
  - *Response: We acknowledge this as a limitation of the study due to lack of data that was available at the health facility level, and lack of integrated data systems making it impossible to match health facility data across different databases. This is highlighted in the Discussion section. The authors believe that this is not peculiar to Pakistan and that systematic sub-national supply chain and programmatic performance capture and reliability of such data is challenging in similar countries. This study is however an important step in moving the supply chain contribution to programme outcomes discussion forward. Our recommendation is that this analysis should be extended to the health facility and community level where data is available, as outlined in the Discussion section. For Pakistan, as a first step, we think it is still valuable to identify districts and then be able to deep-dive for the specific districts.*
- SC Metric 2: Does not consider Cold chain equipment and uses averages.
  - *Response: Request to the reviewer to clarify, as this metric is related to resupply distance and not cold chain storage. No average was used.*
- SC Metric 3: Does not control for variances in stock policies.
  - *Response: In Pakistan, there were no variances in stock policies for districts to the best of our knowledge.*

**3) Are sufficient details provided to allow replication of the method development and its use by others?**

- Some of the assumptions and targets for the calculations are missing.
  - *Response: Please let us know the assumptions and targets that are missing, so we add them appropriately. The targets for all metrics and calculations are presented in the Supply Chain Metrics section, and in Figure 1.*

**4) If any results are presented, are all the source data underlying the results available to ensure full reproducibility?**

- It is not clear how the metrics were derived and how some of the results were calculated.
  - *Response: Request to reviewer to provide more details, so we are able to add more specific details. The Supply Chain Metrics section outlines how each of these metrics was derived and validated and presented in Figure 1. The data for each of the metrics is also available with the paper.*
- Considering equity certainly brings a new dimension to supply chain design. Although I would be cautious to deem the exercise in Pakistan as supply chain design but rather iSC optimization as it did not consider the entire health supply chain, which would further equity goals under UHC.
  - *Response: The approach to supply chain design is based on the direction from the government, in this case the Government of Pakistan. We assessed and recommended changes to supply chain design based on direction from the federal and provincial government. Network optimization for supply chains usually uses specific modeling tools, which were also used in Pakistan. However, the authors went further than looking at the modeling results to look at comprehensive supply chain design.* 
  - *The specific distinction between SC design and iSC optimization is misapplied here. The analysis presented here considers the system’s design and applied an equity lens using three metrics. From experience in multiple countries, System Design analysis could be a country-wide/entire supply chain or a specific scope (e.g. last mile, outreaches, cold chain utilization etc.).*
- The deductions drawn from the analysis and the lack of data at service delivery level leads to inconclusive results. The use of averages, particularly for distances is problematic when analyzing equity and I would suggest a categorization of distances rather.
  - *Response: Refer to above for clarification of the use of averages. Averages was used as a form of categorizing findings. We see this work as a Methods paper, to further the conversation on equity in this area. As this methodology is repeated in other countries and refined, we would anticipate seeing trends that are helpful.*
- The analysis of equity in conflict zones is another opportunity this paper could have addressed in Pakistan.
  - *Response: This was not an ask by the government of Pakistan, and the data on this is not available*

# Peer review: Reviewer response for version 1

**DOI:** 10.21956/gatesopenres.14300.r28753

**Published:** 2020-04-20

**Authors:** Wendy Prosser, Cary Spisak

**Affiliations:** 1 John Snow, Inc., Seattle, WA, USA

General comments:

 This article is very timely as it reflects the global interest in equity in immunization programs and the goal to have more equitable coverage rates across all countries. This is also timely as the article reflects the desire to have more evidence linking supply chain performance to immunization coverage. The two are intuitively linked, as vaccines must be available at service delivery points in order for vaccines to be administered. However, the evidence definitely showing that link is scant.

 This article is a considerable first step to move forward the conversation linking supply chain, coverage and equity. The article does a thorough job of highlighting the limitations and challenges with this type of analysis, which may have prevented finding any clear trends or links between supply chain and coverage equity.

 The metrics as defined by the methodology are judicious and are an innovative twist on standard supply chain indicators in use. It is disappointing that the results of the analysis were either ambiguous or showed no overarching trend or pattern between the chosen supply chain metrics and coverage. The article dutifully addresses this in the limitations, noting the many influential factors on coverage rates and inequity. Because of this ambiguity in the link between supply chain and equity, it would benefit the article to adjust some language to be less definitive about these ambiguous links for the results of each metric (more details on suggestions to address this are listed below).

 The Conclusions don’t necessarily match the results as the analysis showed no notable relationships between the supply chain metrics and immunization coverage. It almost risks narrowing the vision of the article to interpret the results based on expected outcomes. However, as the article notes, the analysis contributed to alternative supply chain configurations that can reduce the distance to resupply points, which does benefit health workers, the supply chain, and, presumably coverage. It is noteworthy to see this thoughtful analytical approach to better supply chain design, and we hope other countries would adapt similar evidence-based approaches to supply chain design.

 Despite the limitations, this article forces the necessary conversation linking supply chain and coverage when making decisions about the supply chain design. The article provides examples of decisions related to the design of the supply chain, for example average hours saved if direct delivery was in place, that could increase the hours a healthcare worker has to provide care for children. As the article marginally notes, this is one of many factors that can influence coverage and equity.

 A noted limitation was the data availability and not being able to analyze the metrics at the facility level and instead using the district level for analysis. This is particularly hindering for cold chain capacity as it may be masking capacity variability among service delivery points. These reviewers agree with the recommendation that using this methodology at the facility level would provide further insight and potentially stronger demonstration of the influence of supply chain design on coverage and equity.

Questions for clarification:

 In the methods (paragraph 1), how do you define “demand” for vaccines as there are many influential factors to true demand? Or is this referring to estimated need?

 Please clarify if CCE considered in this analysis is only used for vaccines.

Specific comments:

 The last sentence in the abstract states that the analysis “highlighted supply chain contributions to program performance.” This reviewer’s understanding of the results is this contribution was not clearly demonstrated although this methodology clearly informed future health system investments and guided decision making for those investments.

 The first sentence of the Conclusion notes that design is often based on cost-efficiency. It is important to note, however, that cost is also an indicator or proxy for different aspects of system performance, such as distance driven. Another notable design influencer is administrative structure of the government and system.

 Specific instances of definitive language that could be adjusted to factor in the ambiguity:

- Last sentence of introduction section: suggest a re-write of this sentence to state this as more of a hypothesis of what this approach aimed to do. As it is currently written, it assumes a connection between design and increasing equity, which the analysis does not definitively determine.
- Results section, “Comparing cold chain capacity and immunization coverage.” The article notes that the relationship between these metrics is ambiguous. The statement in the paragraph on disparities in urban-rural coverage rates, “which might be a result of disparities in cold chain distribution within the district”, is not necessarily based on the results of the analysis which are ambiguous. Suggest re-writing this sentence to recognize the many influential factors in coverage, which are dutifully noted in the introduction of the article.
- Results section, “Comparing weighted average resupply distance to health facilities and immunization coverage.” The average resupply distance in Balochistan, KP and Punjab and relatively similar, yet the coverage rates are quite different. As you state for Balochistan, other factors underpin low coverage. Similarly, other factors underpin high coverage in the other two provinces. How the paragraph is written is biasing the analysis to the ambiguous influence of the supply chain. Suggest a re-write of those two related sentences.

Is the rationale for developing the new method (or application) clearly explained?

Yes

Is the description of the method technically sound?

Yes

Are the conclusions about the method and its performance adequately supported by the findings presented in the article?

Partly

If any results are presented, are all the source data underlying the results available to ensure full reproducibility?

Yes

Are sufficient details provided to allow replication of the method development and its use by others?

Yes

Reviewer Expertise:

NA

We confirm that we have read this submission and believe that we have an appropriate level of expertise to confirm that it is of an acceptable scientific standard.
